# Emerging Strategies and Effective Prevention Measures for Investigating the Association Between Stroke and Sudden Cardiac Fatality

**DOI:** 10.2174/011573403X259676231222053709

**Published:** 2024-01-19

**Authors:** H. Nithesh Kumar, S. Jeevanandham, M. Shankar Ganesh, M. Ashmi Sabana, P. Manivasakam

**Affiliations:** 1 Pharmacy Practice, JKKN College of Pharmacy, Namakkal, India;; 2 Department of Pharmaceutics, Vellalar College of Pharmacy, Erode, India

**Keywords:** Stroke, cardiac death, emerging strategies, wearable devices, genetic testing, stem cell therapy, gene therapy, precision medicine

## Abstract

Stroke-related cardiac death is a significant concern for patients with stroke and their healthcare providers. It is a complex and multifaceted condition that requires careful management of both modifiable and non-modifiable risk factors. This review provides an overview of the pathophysiology, risk factors, and prevention strategies for stroke-related cardiac death. The review highlights the importance of identifying and managing modifiable risk factors such as hypertension, diabetes, and lifestyle factors, as well as non-modifiable risk factors such as age and genetics. Additionally, the review explores emerging strategies for prevention, including the use of wearable devices and genetic testing to identify patients at risk, stem cell therapy and gene therapy for cardiac dysfunction, and precision medicine for personalized treatment plans. Despite some limitations to this review, it provides valuable insights into the current understanding of stroke-related cardiac death and identifies important areas for future research. Ultimately, the implementation of evidence-based prevention strategies and personalized treatment plans has the potential to improve outcomes for patients with stroke and reduce the burden of stroke-related cardiac death in the population.

## 1 INTRODUCTION

Two of the leading causes of death globally are stroke and cardiovascular disease, which place a significant strain on public health systems. An annual budget of $260.7 billion is set aside in the United States alone to treat heart and cerebrovascular illnesses [1, 2]. Many predisposing factors, such as obesity, diabetes, hypertension, high cholesterol, and a family history of heart disease, are shared by these two illnesses [3-6]. Surprisingly, between 2% and 6% of cardiac deaths occur about three months after a brain infarction, indicating a strong correlation between stroke and sudden cardiac death [7, 8].

Emerging evidence underscores the intricate relationship between ischemic stroke and cardiac complications. Notably, studies have revealed that following an ischemic stroke, the patient's body experiences an upsurge in plasma catecholamines and cardiac enzymes, coinciding with elevated levels of troponin and creatine phosphokinase, which signal cardiac cell stress or death [7, 8]. Furthermore, it has been determined that inflammation after an ischemic stroke is a pathogenic route that links the brain and the heart. In stroke patients, C-reactive protein (CRP) has emerged as a critical component linked to the risk of subsequent cardiovascular events [9-12]. The importance of inflammation in the transfer of toxic chemicals from the stroke-affected brain to the circulatory system is highlighted by these findings.

Although there is strong evidence connecting cardiac and cerebrovascular events, these relationships have occasionally been disregarded by the medical profession. Unfortunately, within four weeks of having an ischemic stroke, up to 88% of patients have cardiac symptoms, highlighting how critical it is to identify and treat these correlations [13]. Ischemic injury in the insular cortex, an area of the brain linked to a greater risk of cardiac mortality than other brain regions, is especially concerning [13]. Whether a sub-region or the entire insular cortex is responsible for myocyte death in the heart, however, is still up for debate.

Given the apparent association between ischemic stroke and cardiac failure, it is critical to examine the relationship between compromised cardiac myocytes and the death of brain cells that follow an ischemic stroke. It is necessary to create novel approaches and practical preventative actions in order to comprehend and handle this complex interaction better [14].

An overview of the pathophysiological causes, risk factors, and preventative measures for stroke-related cardiac mortality is intended to be provided by this review article. We will also go through new approaches and best practices for avoiding cardiac mortality following stroke, such as the use of individualized treatment plans, targeted medicines, and innovative diagnostic technologies. We can enhance the prognosis of individuals who are in danger by comprehending the connection between stroke and cardiac mortality and putting into practice efficient preventative measures.

### 1.1 Epidemiology of Stroke and Cardiac Death

The Global Stroke Factsheet, published in 2022, states that stroke ranks as the second most common cause of death globally and the top cause of disability. Over the past 17 years, there has been a 50% rise in the lifetime chance of having a stroke; now, 1 in 4 people are expected to have one throughout their lifetime [15]. The incidence, prevalence, and Disability Adjusted Life Years (DALY) of stroke have all increased significantly between 1990 and 2019. Most stroke cases worldwide occur in lower-income and lower-middle-income nations [16].

The World Health Organization reports that the greatest cause of mortality worldwide is cardiovascular illnesses, or CVDs. Estimates indicate that 17.9 million deaths globally in 2019—or 32% of all fatalities—were related to CVDs. A heart attack or stroke was the cause of 85% of these fatalities [17]. With an estimated prevalence of 2–3 instances per 1000 persons annually in the general population, sudden cardiac death (SCD) is another major factor contributing to cardiovascular mortality. Certain subgroups, such as individuals with a history of heart illness, heart failure, or cardiac arrhythmias, have a greater risk of sickle cell disease (SCD) [18].

### 1.2 Mechanisms of Stroke-Related Cardiac Death

#### 1.2.1 Pathophysiological Mechanisms of Stroke-related Cardiac Death

##### 1.2.1.1 Neurogenic Stress Cardiomyopathy

Neurogenic heart damage is a complex pathophysiology that includes several pathways, such as continued inflammatory processes following a stroke and disruptions in the hypothalamic-pituitary-adrenal (HPA) axis. This results in an increased workload for the heart, coronary artery microvascular constriction, acute and chronic structural and functional abnormalities, and modifications to the heart's molecular signaling, including altered calcium regulation, endothelial dysfunction, inflammation, fibrosis, oxidative stress, mitochondrial dysfunction, myocyte injury, apoptosis, and impaired gene expression [19-24].

Many elements of the pathophysiology of Neurogenic Stunned Myocardium (NSC) are still poorly understood, even after substantial investigation. But the idea that has gained the greatest traction to explain its genesis centres on sympathetic overstimulation brought on by mental and physical stresses, which results in an excessive release of catecholamines [23, 25]. Adrenoreceptor injury, narrowing of the coronary arteries (microvascular and epicardial), and direct catecholamine toxicity are the results of this. For example, fast spikes in catecholamines following a stroke are shown in experimental models of Subarachnoid Haemorrhage (SAH), and these spikes are correlated with higher levels of creatine kinase-MB and troponin T. The higher incidence and severity of cardiovascular problems in the first three days following a stroke may be explained by this phenomenon [8, 26, 27].

The anomalies in LV wall motion found in NSC may be explained by the distinct distribution of β-adrenergic receptors in the left ventricle, which has a larger density near the apex [28]. This increased sensitivity at the peak is particularly noticeable when there is an excess of catecholamines, notably adrenaline. Catecholamines operate locally at the cellular level, perturbing the calcium balance and changing β-adrenergic signalling, which causes myocardial contraction band necrosis and affects coronary microcirculation. [29-31].

Furthermore, an overabundance of adrenaline can cause β2-adrenergic receptors to switch from their typical stimulatory to inhibitory pathways, which might have a detrimental inotropic impact [32]. Research demonstrating a link between cardiac failure and ischemia events in the insular cortex, particularly the right insular cortex, lends more credence to the role of the brain-heart axis in NSC [33-35]. Because the insular cortex receives blood flow from the middle cerebral artery, recent neuroimaging studies employing functional MRI and PET scans support its role as a major regulator of the autonomic nervous system. This makes the insular cortex particularly sensitive to cerebrovascular illness [36, 37].

Neurohormonal pathways and systemic inflammation, in particular, the overproduction of interleukin-1 (IL-1), seem to be important in the formation of NSCs. Soon after a stroke, cytokines are released, and while their plasma levels return to normal in a few hours to days, the myocardial remodelling they cause might last for months [19, 22, 23]. Furthermore, uncontrollably releasing inflammatory mediators can cause cardiac injury in individuals with severe neurological traumas such as SAH, intracerebral hemorrhage, and acute ischemic stroke due to parasympathetic dysfunction [38]. Recent research suggests that microglia, the brain's resident immune cells, play a crucial role in regulating sympathetic nervous system activity, which is a major contributor to pathological cardiac remodeling [39]. The neurohormonal basis for the massive release of norepinephrine and adrenaline also comes from sympathetic stimulation of the adrenal glands, activation of the HPA axis and release of adrenocorticotropic hormone, and elevated levels of plasma cytokines that influence the HPA axis and cause peripheral arterial vasoconstriction, a sudden increase in left ventricular afterload, and elevated left ventricular end-systolic pressure [40-42].

**Fig. (1):**
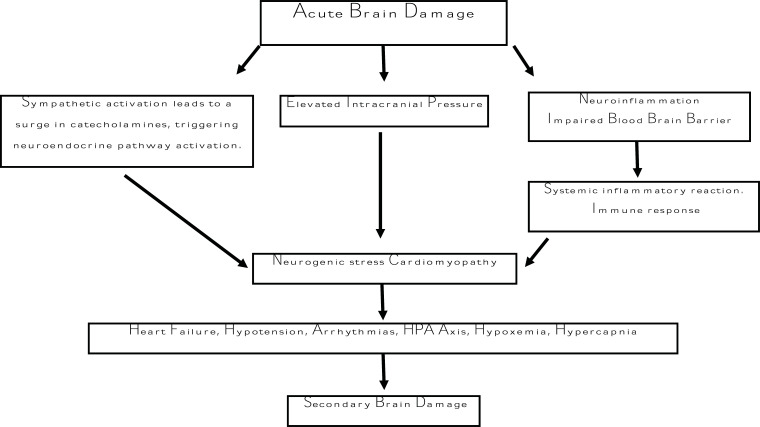
Shows a mechanism of neurogenic stress cardiomyopathy due to stroke.

In order to avoid and manage heart damage in stroke patients, future research focusing on discovering new therapeutic targets, such as β-adrenergic receptor blockade or IL-1 inhibition, along with a comprehensive understanding of the exact pathophysiological processes, may prove to be crucial (Fig. **1**) [43].

##### 1.2.1.2 Cardiac Arrhythmias

Several investigations have demonstrated that acute strokes can cause an imbalance between sympathetic and parasympathetic modulation by interfering with central autonomic function. Myocardial damage, irregular heartbeats, cardiac arrhythmias, and even unexpected death can result from this imbalance. Arrhythmias and cardiac damage after an acute stroke are unlikely to be caused only by underlying or undiscovered cardiac problems, despite the high frequency of coronary artery disease (CAD) and CAD risk factors in acute stroke patients. Acute stroke patients have a pattern of rapidly developing and vanishing ST alterations that are unusual for cardiac reasons, pointing to neurological roots [44].

Autonomic dysfunction is a common occurrence after acute stroke, as evidenced by impaired regulation of heart rate and blood pressure [45]. This includes reduced heart rate variability (HRV) and impaired baroreflex sensitivity (BRS), along with elevated catecholamine and cortisol levels [45]. Decreased HRV has been observed not only in the acute phase of stroke but also at 1 and 6 months post-stroke. The baroreceptor reflex, which stabilizes heart rate and blood pressure during changes in body position, has been found to be reduced in acute stroke patients [46-48]. Excessive catecholamine levels can alter the electrical properties of cardiomyocytes and increase the risk of arrhythmias [49]. Advanced age and the severity of neurological deficits are associated with autonomic instability and heightened sympathetic activity, which can lead to arrhythmias.

When excessive catecholamines overactivate β-adrenergic receptors following an acute stroke, neurogenic heart injury results. This results in the prolonged opening of calcium channels, which hinders the appropriate intracellular calcium ion sequestration that is essential for the relaxation of heart muscle [50]. Extended contraction of the heart muscles may cause damage or even death to the cells. In individuals who have passed away after acute strokes, characteristic lesions such as contraction band necrosis and cardiac myofibrillar degeneration have been seen. These lesions are similar to those observed in hypothalamic stimulation, cardiac muscle reperfusion injuries, and catecholamine infusion [51]. Unlike macrovascular distribution, which is shown in CAD, contraction band necrosis is predominantly prevalent in the distribution of cardiac nerves. It is mostly found in the sub endocardium and may have an impact on the heart's conduction system, raising the possibility of arrhythmias [52]. A syndrome known as “cardiac stunning” with segmental hypokinesis, aberrant ST-segment, and Q-wave patterns can result from transient coronary vasospasm, which is caused by increased sympathetic activity following right-hemispheric ischemic strokes. This condition often goes away in a few days [53]. Excessive catecholamine release after a stroke can also trigger takotsubo cardiomyopathy (TC), which may serve as a substrate for ventricular arrhythmias. Approximately 1.2% of ischemic stroke patients develop TC [53, 54]. This condition is also observed in patients with aneurysmal subarachnoid hemorrhage, characterized by sympathetic surge and myocytolysis.

Insular stroke can interfere with the heart's autonomic control by disrupting the insular cortex located in the lateral sulcus of the brain. Studies on patients with insular stroke have revealed a lower heart rate variability (HRV) and a greater frequency of arrhythmias when compared to age- and sex-matched controls [55]. Electrical stimulation of the insular cortex in animal studies induced significant arrhythmias, similar to those observed after acute strokes [56]. Arrhythmias, autonomic alterations, and lesions in the insular cortex seem to be related. Sympathetic overactivity has been linked to longer repolarization times, lower BRS, and a higher risk of arrhythmogenesis in individuals without CAD [57, 58].

**Fig. (2):**
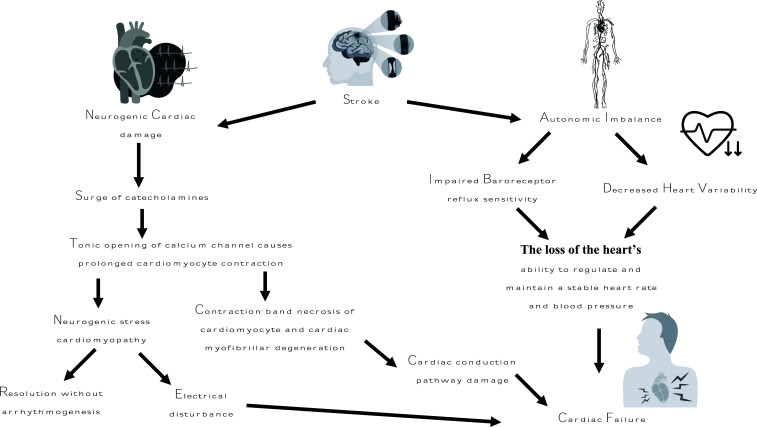
Shows a diagram of how cardiac failure are caused after an ischemic stroke.

Acute stroke-related autonomic imbalance leading to cardiac dysfunction is supported by elevated cardiac biomarkers, not myocardial damage. In a research, the levels of creatine kinase, creatine kinase-MB, myoglobin, and troponin T were raised in the initial days after acute ischemic strokes; however, troponin T, which is a more sensitive marker of myocardial damage, stayed within the reference range. Nonetheless, 7% of patients with ischemic stroke who had no known heart condition or kidney illness had higher troponin T levels, according to bigger research. According to a meta-analysis of 15 studies, 18% of individuals who had acute ischemic or hemorrhagic strokes also had increased levels of troponin T or troponin I. In acute stroke patients, elevated troponin T levels have been linked to higher morbidity and death at 30 days and 6 months [59]. According to a recent study, patients suffering from acute ischemic stroke should have their high-sensitivity cardiac troponin (cTn) levels monitored. It was also suggested that the pattern of acute or chronic elevation of cTn levels, in conjunction with clinical conditions, could aid in the classification of the various causes of elevated cTn levels, such as myocardial infarction, noncoronary causes, and neurogenic heart syndrome (Fig. **2**) [60].

##### 1.2.1.3 Myocardial Infarction

It is still not fully known how exactly a stroke causes myocardial damage and irregularities in the ECG. The HPA, immunological reactions, and other elements including inflammation and intestinal dysbiosis are the main processes. Cardiomyocyte metabolism changes when these systems are disturbed [61, 62].

#### 1.2.2 Autonomic Dysfunction

Changes in autonomic balance are central to a popular theory explaining myocardial damage after a stroke. Between the brain and the heart, the central nervous system controls autonomic reactions, which can be both normal and abnormal [63]. After an acute stroke, alterations in central structures may have a direct impact on the autonomic nervous system (ANS) and cause an overabundance of sympathetic reactions. Pre- and post-ganglionic sympathetic neurons mediate this relationship. The release of intracellular calcium from the sarcoplasmic reticulum is the result of cyclic adenosine monophosphate protein kinase A signalling, which is triggered by excessive sympathetic stimulation that activates βreceptors. This erratic release of calcium into cells causes adenosine triphosphate (ATP) depletion and contractile failure, which in turn causes mitochondrial malfunction and damage to cardiac myocytes that may be reparable or ultimately lead to cell death. The medulla oblongata, nucleus ambiguous, reticular formation, and the nucleus of the nervus vagus are central structures that regulate parasympathetic activity. They release acetylcholine through epicardial ganglion plexuses and postganglionic nerve fibres. Muscarinic receptors regulate parasympathetic activity by decreasing cyclic adenosine monophosphate, which may decrease contractility by delaying depolarization. The adrenal glands, pituitary, and brain make up the HPA axis. After an acute stroke, increased calcium release, ATP depletion, and oxidative stress are caused by the adrenal glands' production of cortisol and activation of α1 adrenoreceptors by catecholamines. This suggests that cardiac toxicity may result from catecholamines released into the bloodstream from myocardial nerve terminals [63].

##### 1.2.2.1 Immune Response

Cerebral hemorrhages and ischemic strokes often trigger a systemic inflammatory response [64]. Damage to plasma membranes from strokes raises ATP levels, which further activates microglia and encourages the synthesis of inflammatory cytokines such IL-2, IL-6, myeloperoxidase, and integrins. These cytokines then promote oxidative stress [65]. In atherosclerotic plaques, inflammatory cytokines build up on endothelial cells and degrade collagen. A weakening of the fibrous membranes may cause a heart attack [66].

##### 1.2.2.2 Other Contributing Factors

Additional mechanisms include interactions among intestinal flora, the central nervous system (CNS), and the cardiovascular system. Some studies have indicated that patients experience disruption in intestinal permeability after an acute stroke [67]. Modified intestinal permeability allows bacteria and endotoxins to enter the circulation, which can exacerbate cardiac injury by increasing pro-inflammatory cytokines and systemic inflammation. Moreover, blood metabolites including trimethylamine oxide and indoxyl sulphate are affected by the translocation of bacteria and endotoxins [68]. While trimethylamine-N-oxide is linked to cardiac dysfunction, heart failure, and an increased risk of thrombosis, indoxyl sulphate influences cardiac remodelling through NFk [69].

#### 1.2.3 Relationship between Location and Size of Stroke and Risk of Cardiac Death

The location and size of the stroke can have an impact on the risk of cardiac death. Generally, larger and more severe strokes are associated with a higher risk of cardiac complications, including cardiac death. This is because larger strokes can cause more extensive damage to the brain, leading to a greater release of catecholamines and a higher likelihood of developing cardiac complications.

The specific location of the stroke can also play a role in the risk of cardiac death. For example, strokes in the insular cortex, which is a region deep within the brain, have been associated with a higher risk of cardiac complications. The insular cortex is involved in the regulation of the autonomic nervous system, which controls the heart rate and blood pressure, and damage to this area can disrupt the normal functioning of the heart.

Strokes that affect the left side of the brain have been associated with a higher risk of cardiac complications compared to strokes that affect the right side of the brain. This is because the left side of the brain is responsible for controlling the sympathetic nervous system, which can have an impact on heart function. Overall, the location and size of the stroke can both contribute to the risk of cardiac death, but other factors such as pre-existing cardiovascular disease and risk factors also play a role. Overall, the complex interplay between stroke and cardiac dysfunction highlights the need for a comprehensive approach to prevent stroke-related cardiac death. By understanding the mechanisms underlying this link and identifying individuals at high risk, clinicians can develop targeted prevention strategies to reduce the risk of cardiac complications and sudden death in patients with stroke [70].

### 1.3 Risk Factors for Stroke-related Cardiac Death

#### 1.3.1 Modifiable Risk Factors (e.g., Hypertension, Diabetes, Smoking, Obesity)

- Hypertension (high blood pressure) is a major risk factor for both stroke and heart disease. Over time, high blood pressure can damage the arteries, making them less flexible and more prone to blockages that can cause heart attacks and strokes.
- Diabetes is another significant risk factor for both stroke and heart disease. People with diabetes have higher levels of blood sugar, which can damage blood vessels and increase the risk of developing cardiovascular disease.
- Smoking is a major risk factor for both stroke and heart disease because it can damage the lining of blood vessels, increase the buildup of plaque in the arteries, and increase the risk of blood clots.
- Obesity is also a significant risk factor for stroke and heart disease. Being overweight or obese can lead to the development of high blood pressure, high cholesterol levels, and diabetes, all of which increase the risk of cardiovascular disease.
- Addressing these modifiable risk factors through lifestyle changes such as quitting smoking, maintaining a healthy weight, exercising regularly, and managing hypertension and diabetes can help to reduce the risk of both stroke and cardiac death [71].

#### 1.3.2 Non-modifiable Risk Factors Include

- Age: The risk of stroke and cardiac death increases with age, and the incidence of stroke-related cardiac death is highest in those over the age of 65.
- Gender: Men have a higher risk of stroke-related cardiac death than women.
- Genetics: A family history of stroke and heart disease can increase the risk of stroke-related cardiac death.
- Race and ethnicity: African Americans and Hispanic Americans have a higher risk of stroke and stroke-related cardiac death than non-Hispanic whites.
- Prior stroke: Individuals who have had a prior stroke are at higher risk of stroke-related cardiac death than those who have not [72, 73].

#### 1.3.3 Role of Comorbidities (e.g., Atrial Fibrillation, Heart Failure) in Increasing the Risk of Stroke-related Cardiac Death

Comorbidities such as atrial fibrillation and heart failure can significantly increase the risk of stroke-related cardiac death. Atrial fibrillation, a type of irregular heartbeat, is a major risk factor for ischemic stroke and can increase the risk of stroke-related cardiac death by up to five-fold. Similarly, heart failure, a condition in which the heart is unable to pump enough blood to meet the body's needs, can lead to the development of blood clots that can cause stroke and increase the risk of cardiac death. Other comorbidities that can increase the risk of stroke-related cardiac death include coronary artery disease, peripheral artery disease, and valvular heart disease. These conditions can contribute to the development of atherosclerosis, a buildup of plaque in the arteries that can increase the risk of stroke and cardiac death. Overall, the presence of comorbidities can significantly increase the risk of stroke-related cardiac death and should be carefully managed and monitored in patients who have experienced a stroke [74, 75].

### 1.4 Prevention Strategies for Stroke-related Cardiac Death

#### 1.4.1 Primary Prevention Strategies to Reduce the Risk of Stroke and Cardiac Death (e.g., Lifestyle Modifications, Medications)

##### 1.4.1.1 Lifestyle Modifications

- Healthy diet: A healthy diet that is low in saturated and trans fats, and high in fruits, vegetables, whole grains, and lean protein sources can help reduce the risk of stroke and cardiac death. The DASH (Dietary Approaches to Stop Hypertension) diet has been shown to be particularly effective in reducing the risk of stroke.
- Regular exercise: Regular physical activity, such as brisk walking, cycling, or swimming, can help improve cardiovascular health and reduce the risk of stroke and cardiac death.
- Smoking cessation: Smoking is a major risk factor for stroke and cardiac death. Quitting smoking can significantly reduce the risk of these conditions.
- Moderate alcohol consumption: Moderate alcohol consumption (up to one drink per day for women and up to two drinks per day for men) may have a protective effect against stroke and cardiac death. However, excessive alcohol consumption can increase the risk of these conditions [76, 77].

##### 1.4.1.2 Medications

- Blood pressure control: High blood pressure is a major risk factor for both stroke and cardiac death. Medications that lower blood pressure, such as ACE inhibitors, ARBs, and diuretics, can help reduce the risk of these conditions.
- Cholesterol management: High cholesterol levels can increase the risk of stroke and cardiac death. Statins are a class of medications that can help lower cholesterol levels and reduce the risk of these conditions.
- Antiplatelet therapy: Antiplatelet medications, such as aspirin or clopidogrel, can help prevent blood clots and reduce the risk of stroke.
- Anticoagulation therapy: In patients with atrial fibrillation, anticoagulation therapy with medications such as warfarin or newer oral anticoagulants can help reduce the risk of stroke [78].

#### 1.4.2 Secondary Prevention Strategies to Reduce the Risk of Recurrent Stroke and Cardiac Death (e.g., Anticoagulation Therapy, Blood Pressure Control, Cholesterol-lowering Medications)

- Anticoagulation therapy: For individuals with certain types of strokes, such as ischemic stroke caused by atrial fibrillation, anticoagulation therapy may be recommended to reduce the risk of blood clots and subsequent stroke or cardiac death.
- Blood pressure control: High blood pressure is a major risk factor for stroke and cardiac death. Controlling blood pressure through lifestyle modifications such as exercise and dietary changes, as well as medications, can significantly reduce the risk of these events.
- Cholesterol-lowering medications: High levels of LDL cholesterol can increase the risk of stroke and cardiac death. Medications such as statins can help lower cholesterol levels and reduce this risk.
- Smoking cessation: Smoking is a major risk factor for both stroke and cardiac death. Quitting smoking can significantly reduce the risk of these events.
- Regular exercise: Regular exercise can help improve cardiovascular health and reduce the risk of stroke and cardiac death.
- Dietary modifications: A diet rich in fruits, vegetables, whole grains, lean proteins, and low in saturated and trans fats can help reduce the risk of stroke and cardiac death.
- Management of comorbidities: Comorbidities such as atrial fibrillation and heart failure can increase the risk of stroke and cardiac death. Proper management of these conditions through medication and lifestyle modifications can help reduce this risk [79, 80].

#### 1.4.3 Importance of Early Identification and Management of Cardiac Risk Factors in Patients with Stroke

Early identification and management of cardiac risk factors in patients with stroke is crucial to prevent stroke-related cardiac death. It is well-established that patients with stroke are at increased risk of developing cardiovascular complications, including cardiac arrhythmias, heart failure, and sudden cardiac death. Therefore, it is important to screen for and manage modifiable risk factors such as hypertension, diabetes, smoking, and obesity, which can significantly increase the risk of these complications. Additionally, the presence of comorbidities such as atrial fibrillation and heart failure can further increase the risk of stroke-related cardiac death. These comorbidities should also be identified and managed in a timely manner to reduce the risk of adverse outcomes. Early management of cardiac risk factors can be achieved through a combination of lifestyle modifications, such as regular exercise, healthy diet, and smoking cessation, as well as appropriate pharmacological interventions, including anticoagulation therapy, blood pressure control, and cholesterol-lowering medications. By implementing these measures early on, healthcare providers can significantly reduce the risk of recurrent stroke and cardiac death in patients with stroke [81, 82].

### 1.5 Emerging Strategies for Prevention of Stroke-related Cardiac Death

#### 1.5.1 Use of Novel Diagnostic Tools (e.g., Wearable Devices, Genetic Testing) to Identify Patients at Risk for Cardiac Death after Stroke

- Wearable devices: The use of wearable devices such as smartwatches and fitness trackers may help to identify patients at risk for stroke-related cardiac death by monitoring heart rate, rhythm, and activity levels. For example, some devices can alert users to irregular heart rhythms that may be indicative of atrial fibrillation, a major risk factor for stroke-related cardiac death. Wearable devices may also help patients monitor their blood pressure and track their physical activity levels, which can help to reduce their risk of recurrent stroke and cardiac events [83, 84].
- Genetic testing: Recent advances in genetic testing have led to the identification of several genetic variants that are associated with an increased risk of stroke-related cardiac death. For example, mutations in the SCN5A gene have been linked to an increased risk of sudden cardiac death, particularly in patients with ischemic stroke. Genetic testing may help to identify patients who are at particularly high risk for cardiac death after stroke, allowing for targeted prevention strategies such as early initiation of anticoagulation therapy or implantation of an implantable cardioverter defibrillator (ICD) [85-87].
- Artificial intelligence: Artificial intelligence (AI) algorithms may help to identify patients at risk for stroke-related cardiac death by analyzing large amounts of clinical data and identifying patterns that are indicative of increased risk. For example, AI algorithms may be able to identify patients with a high risk of atrial fibrillation based on their ECG patterns, allowing for early detection and intervention. AI may also be used to predict the risk of recurrent stroke and cardiac events based on a patient's medical history, imaging studies, and other clinical data, allowing for personalized prevention strategies [88, 89].

#### 1.5.2 Development of Targeted Therapies (e.g., Stem Cell Therapy, Gene Therapy) for Cardiac Dysfunction after Stroke

Stem cell therapy and gene therapy are two promising areas of research that may help prevent cardiac dysfunction and reduce the risk of cardiac death after stroke.

- Stem cell therapy involves the transplantation of stem cells into the damaged heart tissue to regenerate healthy tissue and improve cardiac function. Several preclinical and clinical studies have shown the potential benefits of stem cell therapy in reducing cardiac damage and improving cardiac function after stroke. However, further research is needed to establish its efficacy and safety in humans [90-92].
- Gene therapy involves the introduction of genes into the cells to treat or prevent diseases. It has the potential to address the underlying genetic causes of cardiac dysfunction after stroke. Several preclinical studies have shown promising results of gene therapy in improving cardiac function after stroke. However, more research is needed to assess its safety and efficacy in humans. While these emerging strategies hold promise, further research is needed to determine their safety and effectiveness in preventing stroke-related cardiac death [93, 94].

#### 1.5.3 Use of Precision Medicine to Develop Personalized Treatment Plans for Patients at Risk for Stroke-related Cardiac Death

A developing method for disease prevention and treatment is precision medicine, which considers a person's unique genes, environment, and lifestyle. The use of precision medicine in stroke-related cardiac death involves identifying specific genetic, biochemical, and clinical factors that influence an individual's risk of developing cardiac complications after stroke. This approach can help develop personalized treatment plans for patients at risk for stroke-related cardiac death. For example, studies have shown that genetic variations can influence the response to anticoagulation therapy and increase the risk of bleeding complications. By identifying these genetic variations, healthcare providers can tailor anticoagulation therapy to individual patients to reduce the risk of both stroke and bleeding complications. Similarly, genetic testing can identify patients with an increased risk of developing atrial fibrillation after stroke, allowing for earlier detection and treatment of this common comorbidity [95].

Precision medicine can also help identify patients who may benefit from more aggressive blood pressure control or cholesterol-lowering medications. By identifying individuals at higher risk for recurrent stroke or cardiac death, healthcare providers can develop targeted treatment plans to reduce the risk of future events. Overall, the use of precision medicine in stroke-related cardiac death has the potential to improve outcomes and reduce the burden of stroke-related complications by tailoring treatment plans to individual patients [96].

## CONCLUSION

Stroke-related cardiac death is a significant concern for patients with stroke and their healthcare providers. The pathophysiological mechanisms, risk factors, and strategies for the prevention of stroke-related cardiac death are complex and multifaceted. This review has highlighted the importance of identifying and managing modifiable and non-modifiable risk factors for stroke-related cardiac death, as well as the emerging strategies for prevention and personalized treatment plans. However, there are limitations to this review, including the limited scope of the literature search and the potential for bias in the selection of studies. Despite these limitations, this review has provided valuable insights into the current understanding of stroke-related cardiac death and identified important areas for future research. Ultimately, the implementation of evidence-based prevention strategies and personalized treatment plans has the potential to improve the outcomes of patients with stroke and reduce the burden of stroke-related cardiac death in the population.

## LIST OF ABBREVIATIONS

CRP: C-reactive Protein
DALY: Disability Adjusted Life Years
SCD: Sudden Cardiac Death
HPA: Hypothalamic-pituitary-adrenal
NSC: Neurogenic Stunned Myocardium

## References

[1] (2011). Centers for Disease Control and Prevention (CDC). Prevalence of coronary heart disease--United States, 2006-2010.. MMWR Morb. Mortal. Wkly. Rep..

[2] (2012). Centers for Disease Control and Prevention (CDC). Prevalence of stroke--United States, 2006-2010.. MMWR Morb. Mortal. Wkly. Rep..

[3] Kannel W.B. (2000). The Framingham Study: ITS 50-year legacy and future promise.. J. Atheroscler. Thromb..

[4] Lo E.H., Dalkara T., Moskowitz M.A. (2003). Mechanisms, challenges and opportunities in stroke.. Nat. Rev. Neurosci..

[5] Zhang Z.G., Chopp M. (2009). Neurorestorative therapies for stroke: Underlying mechanisms and translation to the clinic.. Lancet Neurol..

[6] Lawlor D.A., Smith G.D., Leon D.A., Sterne J.A.C., Ebrahim S. (2002). Secular trends in mortality by stroke subtype in the 20th century: A retrospective analysis.. Lancet.

[7] Oppenheimer S.M., Hachinski V.C. (1992). The cardiac consequences of stroke.. Neurol. Clin..

[8] Prosser J., MacGregor L., Lees K.R., Diener H.C., Hacke W., Davis S. (2007). Predictors of early cardiac morbidity and mortality after ischemic stroke.. Stroke.

[9] Klingelhöfer J., Sander D. (1997). Cardiovascular consequences of clinical stroke.. Baillieres Clin. Neurol..

[10] Adams J.E., Abendschein D.R., Jaffe A.S. (1993). Biochemical markers of myocardial injury. Is MB creatine kinase the choice for the 1990s?. Circulation.

[11] Snider S.R., Kuchel O. (1983). Dopamine: An important neurohormone of the sympathoadrenal system. Significance of increased peripheral dopamine release for the human stress response and hypertension.. Endocr. Rev..

[12] Di Napoli M., Papa F. (2002). Inflammation, hemostatic markers, and antithrombotic agents in relation to long-term risk of new cardiovascular events in first-ever ischemic stroke patients.. Stroke.

[13] Ay H., Koroshetz W.J., Benner T. (2006). Neuroanatomic correlates of stroke-related myocardial injury.. Neurology.

[14] Ishikawa H., Tajiri N., Vasconcellos J. (2013). Ischemic stroke brain sends indirect cell death signals to the heart.. Stroke.

[15] (2022). World Stroke Day.. http://www.who.int.

[16] Thayabaranathan T., Kim J., Cadilhac D.A. (2022). Global stroke statistics 2022.. Int. J. Stroke.

[17] (2021). World Health Organization.. https://www.who.int/news-room/factsheets/detail/cardiovascular-diseases-(cvds).

[18] Hayashi M., Shimizu W., Albert C.M. (2015). The spectrum of epidemiology underlying sudden cardiac death.. Circ. Res..

[19] Sposato L.A., Fridman S., Whitehead S.N., Lopes R.D. (2018). Linking stroke-induced heart injury and neurogenic atrial fibrillation: A hypothesis to be proven.. J. Electrocardiol..

[20] Sposato L.A., Hilz M.J., Aspberg S. (2020). Post-stroke cardiovascular complications and neurogenic cardiac injury.. J. Am. Coll. Cardiol..

[21] Templin C., Ghadri J.R., Diekmann J. (2015). Clinical features and outcomes of takotsubo (Stress) cardiomyopathy.. N. Engl. J. Med..

[22] Scheitz J.F., Nolte C.H., Doehner W., Hachinski V., Endres M. (2018). Stroke–heart syndrome: Clinical presentation and underlying mechanisms.. Lancet Neurol..

[23] Pelliccia F., Kaski J.C., Crea F., Camici P.G. (2017). Pathophysiology of takotsubo syndrome.. Circulation.

[24] Balint B., Jaremek V., Thorburn V., Whitehead S.N., Sposato L.A. (2019). Left atrial microvascular endothelial dysfunction, myocardial inflammation and fibrosis after selective insular cortex ischemic stroke.. Int. J. Cardiol..

[25] Suzuki H., Matsumoto Y., Kaneta T. (2014). Evidence for brain activation in patients with takotsubo cardiomyopathy.. Circ. J..

[26] Masuda T., Sato K., Yamamoto S. (2002). Sympathetic nervous activity and myocardial damage immediately after subarachnoid hemorrhage in a unique animal model.. Stroke.

[27] Kallmünzer B., Breuer L., Kahl N. (2012). Serious cardiac arrhythmias after stroke: Incidence, time course, and predictors: A systematic, prospective analysis.. Stroke.

[28] Jensen J.K., Ueland T., Aukrust P. (2012). Highly sensitive troponin T in patients with acute ischemic stroke.. Eur. Neurol..

[29] Lyon A.R., Rees P.S.C., Prasad S., Poole-Wilson P.A., Harding S.E. (2008). Stress (Takotsubo) cardiomyopathy a novel pathophysiological hypothesis to explain catecholamine-induced acute myocardial stunning.. Nat. Clin. Pract. Cardiovasc. Med..

[30] Ghadri J.R., Wittstein I.S., Prasad A. (2018). International expert consensus document on takotsubo syndrome (Part I): Clinical characteristics, diagnostic criteria, and pathophysiology.. Eur. Heart J..

[31] White M., Wiechmann R.J., Roden R.L. (1995). Cardiac beta-adrenergic neuroeffector systems in acute myocardial dysfunction related to brain injury. Evidence for catecholamine-mediated myocardial damage.. Circulation.

[32] Paur H., Wright P.T., Sikkel M.B. (2012). High levels of circulating epinephrine trigger apical cardiodepression in a β2-adrenergic receptor/Gi-dependent manner: A new model of Takotsubo cardiomyopathy.. Circulation.

[33] Jung J.M., Kim J.G., Kim J.B. (2016). Takotsubo like myocardial dysfunction in ischemic stroke.. Stroke.

[34] Yoshimura S., Toyoda K., Ohara T. (2008). Takotsubo cardiomyopathy in acute ischemic stroke.. Ann. Neurol..

[35] Bleilevens C., Roehl A.B., Zoremba N., Tolba R., Rossaint R., Hein M. (2015). Insular infarct size but not levosimendan influenced myocardial injury triggered by cerebral ischemia in rats.. Exp. Brain Res..

[36] Nagai M., Hoshide S., Kario K. (2010). The insular cortex and cardiovascular system: A new insight into the brain-heart axis.. J. Am. Soc. Hypertens..

[37] Craig A.D. (2009). How do you feel now? The anterior insula and human awareness.. Nat. Rev. Neurosci..

[38] Mashaly H.A., Provencio J.J. (2008). Inflammation as a link between brain injury and heart damage: The model of subarachnoid hemorrhage.. Cleve. Clin. J. Med..

[39] Wang X., Cui L., Ji X. (2022). Cognitive impairment caused by hypoxia: From clinical evidences to molecular mechanisms.. Metab. Brain Dis..

[40] Tavazzi G., Zanierato M., Via G., Iotti G.A., Procaccio F. (2017). Are neurogenic stress cardiomyopathy and takotsubo different syndromes with common pathways?. JACC Heart Fail..

[41] Fassbender K., Schmidt R., Mössner R., Daffertshofer M., Hennerici M. (1994). Pattern of activation of the hypothalamic-pituitary-adrenal axis in acute stroke. Relation to acute confusional state, extent of brain damage, and clinical outcome.. Stroke.

[42] Sobowale O.A., Parry-Jones A.R., Smith C.J., Tyrrell P.J., Rothwell N.J., Allan S.M. (2016). Interleukin-1 in Stroke.. Stroke.

[43] Edwards A.V., Jones C.T. (1993). Autonomic control of adrenal function.. J. Anat..

[44] Oppenheimer S., Norris J.W. (1995). Neurology and General Medicine: the Neurological Aspects of Medical Disorder..

[45] Myers M.G., Norris J.W., Hachniski V.C., Sole M.J. (1981). Plasma norepinephrine in stroke.. Stroke.

[46] Christensen H., Boysen G., Johannesen H.H. (2004). Serum-cortisol reflects severity and mortality in acute stroke.. J. Neurol. Sci..

[47] Naver H.K., Blomstrand C., Wallin B.G. (1996). Reduced heart rate variability after right-sided stroke.. Stroke.

[48] Korpelainen J.T., Sotaniemi K.A., Huikuri H.V., Myllylä V.V. (1996). Abnormal heart rate variability as a manifestation of autonomic dysfunction in hemispheric brain infarction.. Stroke.

[49] Robinson T.G., James M., Youde J., Panerai R., Potter J. (1997). Cardiac baroreceptor sensitivity is impaired after acute stroke.. Stroke.

[50] Mikolich J.R., Jacobs W.C., Fletcher G.F. (1981). Cardiac arrhythmias in patients with acute cerebrovascular accidents.. JAMA.

[51] Koppikar S., Baranchuk A., Guzmán J.C., Morillo C.A. (2013). Stroke and ventricular arrhythmias.. Int. J. Cardiol..

[52] Samuels M.A. (2007). The brain-heart connection.. Circulation.

[53] Jacob W., Van Bogaert A., De Groodt-Lasseel M.H. (1972). Myocardial ultrastructure and haemodynamic reactions during experimental subarachnoid haemorrhage.. J. Mol. Cell. Cardiol..

[54] Wang T.D., Wu C.C., Lee Y.T. (1997). Myocardial stunning after cerebral infarction.. Int. J. Cardiol..

[55] Tokgözoglu S.L., Batur M.K., Topçuoglu M.A., Saribas O., Kes S., Oto A. (1999). Effects of stroke localization on cardiac autonomic balance and sudden death.. Stroke.

[56] Oppenheimer S.M., Wilson J.X., Guiraudon C., Cechetto D.F. (1991). Insular cortex stimulation produces lethal cardiac arrhythmias: A mechanism of sudden death?. Brain Res..

[57] Vaseghi M., Zhou W., Shi J. (2012). Sympathetic innervation of the anterior left ventricular wall by the right and left stellate ganglia.. Heart Rhythm.

[58] Volders P.G.A. (2010). Novel insights into the role of the sympathetic nervous system in cardiac arrhythmogenesis.. Heart Rhythm.

[59] Song H.S., Back J.H., Jin D.K. (2008). Cardiac troponin T elevation after stroke: relationships between elevated serum troponin T, stroke location, and prognosis.. J. Clin. Neurol..

[60] Scheitz J.F., Nolte C.H., Laufs U., Endres M. (2015). Application and interpretation of high-sensitivity cardiac troponin assays in patients with acute ischemic stroke.. Stroke.

[61] Christensen H., Boysen G., Christensen A.F., Johannesen H.H. (2005). Insular lesions, ECG abnormalities, and outcome in acute stroke.. J. Neurol. Neurosurg. Psychiatry.

[62] Oppenheimer S. (2006). Cerebrogenic cardiac arrhythmias.. Clin. Auton. Res..

[63] Silvani A., Calandra-Buonaura G., Dampney R.A.L., Cortelli P. (2016). Brain–heart interactions: Physiology and clinical implications.. Philos. Trans.- Royal Soc., Math. Phys. Eng. Sci..

[64] Dhar R., Diringer M.N. (2008). The burden of the systemic inflammatory response predicts vasospasm and outcome after subarachnoid hemorrhage.. Neurocrit. Care.

[65] Vahidy F.S., Parsha K.N., Rahbar M.H. (2016). Acute splenic responses in patients with ischemic stroke and intracerebral hemorrhage.. J. Cereb. Blood Flow Metab..

[66] Tay A., Tamam Y., Yokus B., Ustundag M., Orak M. (2015). Serum myeloperoxidase levels in predicting the severity of stroke and mortality in acute ischemic stroke patients.. Eur. Rev. Med. Pharmacol. Sci..

[67] Chen Z., Venkat P., Seyfried D., Chopp M., Yan T., Chen J. (2017). Brain heart interaction.. Circ. Res..

[68] Lekawanvijit S., Adrahtas A., Kelly D.J., Kompa A.R., Wang B.H., Krum H. (2010). Does indoxyl sulfate, a uraemic toxin, have direct effects on cardiac fibroblasts and myocytes?. Eur. Heart J..

[69] Zhu W., Gregory J.C., Org E. (2016). Gut microbial metabolite tmao enhances platelet hyperreactivity and thrombosis risk.. Cell.

[70] Rincon F., Dhamoon M., Moon Y. (2008). Stroke location and association with fatal cardiac outcomes: Northern Manhattan Study (NOMAS).. Stroke.

[71] (2020). Centers for Disease Control and Prevention.. https://www.cdc.gov/chronicdisease/resources/publications/factsheets/heart-diseasestroke.htm.

[72] Boehme A.K., Esenwa C., Elkind M.S.V. (2017). Stroke risk factors, genetics, and prevention.. Circ. Res..

[73] Ada’s Medical Knowledge Team (2017). Cardiovascular Disease Risk Factors.. https://ada.com/cardiovascular-disease-riskfactors/.

[74] Cipolla M.J., Liebeskind D.S., Chan S.L. (2018). The importance of comorbidities in ischemic stroke: Impact of hypertension on the cerebral circulation.. J. Cereb. Blood Flow Metab..

[75] Nelson M.L.A., McKellar K.A., Yi J. (2017). Stroke rehabilitation evidence and comorbidity: A systematic scoping review of randomized controlled trials.. Top. Stroke Rehabil..

[76] Arnett DK, Blumenthal RS, Albert MA (2019). ACC/AHA Guideline on the Primary Prevention of Cardiovascular Disease.. Circulation.

[77] Mayo Clinic (2019). Top strategies to prevent heart disease. https://www.mayoclinic.org/diseasesconditions/heart-disease/in-depth/heart-diseaseprevention/art-20046502.

[78] (2019). Stroke Prevention: Practice Essentials, Overview, Primary Prevention of Stroke.. https://emedicine.medscape.com/article/323662-overview.

[79] American Nurse: The official Journal of the American Nurses Association (ANA).. https://www.myamericannurse.com/secondaryprevention-of-stroke/.

[80] (2014). Hankey GJ, Secondary stroke prevention.. The Lancet Neurology.

[81] Frerich S., Malik R., Georgakis M.K. (2022). Cardiac risk factors for stroke: A comprehensive mendelian randomization study.. Stroke.

[82] Jauch E.C., Saver J.L., Adams H.P. (2013). Guidelines for the early management of patients with acute ischemic stroke: A guideline for healthcare professionals from the American Heart Association/American Stroke Association.. Stroke.

[83] Wearable Devices for Stroke Prediction.. https://encyclopedia.pub/entry/7512.

[84] Kutyifa V., Moss A.J., Klein H. (2015). Use of the wearable cardioverter defibrillator in high-risk cardiac patients.. Circulation.

[85] Bezzina C.R., Lahrouchi N., Priori S.G. (2015). Genetics of sudden cardiac death.. Circ. Res..

[86] Laitinen-Forsblom P.J., Mäkynen P., Mäkynen H. (2006). SCN5A mutation associated with cardiac conduction defect and atrial arrhythmias.. J. Cardiovasc. Electrophysiol..

[87] Zaklyazminskaya E., Dzemeshkevich S. (2016). The role of mutations in the SCN5A gene in cardiomyopathies.. Biochim. Biophys. Acta Mol. Cell Res..

[88] Krittanawong C., Virk H.U.H., Bangalore S. (2020). Machine learning prediction in cardiovascular diseases: A meta-analysis.. Sci. Rep..

[89] Ding L., Liu C., Li Z., Wang Y. (2020). Incorporating artificial intelligence into stroke care and research.. Stroke.

[90] Tenreiro M.F., Louro A.F., Alves P.M., Serra M. (2021). Next generation of heart regenerative therapies: Progress and promise of cardiac tissue engineering.. NPJ Regen. Med..

[91] Abdelwahid E., Kalvelyte A., Stulpinas A., de Carvalho K.A.T., Guarita-Souza L.C., Foldes G. (2016). Stem cell death and survival in heart regeneration and repair.. Apoptosis.

[92] (2013). Harvard Health Publishing.. https://www.health.harvard.edu/hearthealth/repairing-the-heart-with-stem-cells.

[93] Shimamura M., Nakagami H., Sanada F., Morishita R. (2020). Progress of gene therapy in cardiovascular disease.. Hypertension.

[94] Kieserman J.M., Myers V.D., Dubey P., Cheung J.Y., Feldman A.M. (2019). Current landscape of heart failure gene therapy.. J. Am. Heart Assoc..

[95] Brehm K., Schack J., Heilmann C., Blanke P., Geissler H.J., Beyersdorf F. (2013). Mechanical heart valve recipients: Anticoagulation in patients with genetic variations of phenprocoumon metabolism.. Europ. J. Card. Thorac. Surg..

[96] Petersen N.H., Sheth K.N., Jha R.M. (2023). Precision medicine in neurocritical care for cerebrovascular disease cases.. Stroke.

